# A randomised controlled trial to assess the feasibility of utilising virtual reality to facilitate analgesia during external cephalic version

**DOI:** 10.1038/s41598-020-60040-3

**Published:** 2020-02-21

**Authors:** Vinayak Smith, Ritesh Rikain Warty, Ravi Kashyap, Peter Neil, Carol Adriaans, Amrish Nair, Sathya Krishnan, Fabricio Da Silva Costa, Beverley Vollenhoven, Euan M. Wallace

**Affiliations:** 1 The Ritchie Centre, Department of Obstetrics and Gynaecology, Monash University, 252 Clayton Road, Clayton, 3168 Victoria Australia; 2 Biorithm Pte. Ltd., Block 81, Ayer Rajah Crescent, Unit #03-53, 139967 Singapore, Singapore; 3 Alo VR Pte. Ltd, 3B Teo Hong Road, Singapore, Singapore; 4 Monash Women’s, Monash Health, Monash Medical Centre, Clayton, Monash Health, 252 Clayton Road, Clayton, 3168 Victoria Australia; 5 West Gippsland Health Service, Warragul, 3820 Victoria Australia; 6 Department of Gynecology and Obstetrics, Ribeirão Preto Medical School, University of São Paulo, Ribeirão Preto, São Paulo Brazil

**Keywords:** Randomized controlled trials, Pain

## Abstract

External cephalic version **(**ECV**)** is associated with a moderate degree of pain. Virtual reality **(**VR**)** is a technology that has shown promise in offering procedural analgesia. We undertook a clinical pilot to assess the viability of VR to reduce pain during ECV. In an open randomised controlled trial **(**RCT**)**, we randomised 50 women to either VR or standard care each **(**25 per group**)**. Women receiving VR were administered VR content **(**Skylights**)** via a headset. Pre- and post-procedural measures of pain, anxiety, device experience and vital signs were measured. There were no significant differences between groups **(**VR/no VR**)** in pain scores **(**60.68 vs 49.76; p = 0.2**)**, ECV success rates **(**80% vs 76%; p = 0.7**)** or anxiety levels. The women receiving VR had a significantly higher anticipation of pain pre-procedurally **(**70.0 vs 50.0; p = 0.03**)**. 20 **(**80%**)** of the VR women indicated that they would use VR again and 22 **(**88%**)** indicated they would recommend it to a friend having ECV. There were no significant differences between groups for side effects encountered or changes in vital signs. We have shown that using VR during ECV is feasible and appears safe. Our results inform the design of future RCTs.

## Introduction

Breech presentation at term occurs in about 3–4% of pregnancies and is defined as occurring when the pelvis and/or lower limbs of the fetus are oriented downwards. Choices in the care of women with a breech presentation at term include external cephalic version **(**ECV**)**, caesarean section **(**C-Section**)**, and vaginal breech delivery^1^.

In contrast to C-sections and vaginal breech delivery, ECV is a safer and less interventional approach whereby a fetus is manually rotated to the cephalic **(**head down**)** position by applying pressure to the maternal abdomen. ECV is recommended on the basis that it offers the mother a safer choice of a normal vaginal birth. It has a 60% success rate of cephalic presentation at the onset of labour and decreases the C-Section rate by almost 40%^2^.

Given the purported benefits of the procedure and the lack of alternatives, an argument could be made that it should be offered to all women with a breech presentation. Interestingly, between 1995 and 2001, the number of patients aware of the procedure had significantly increased **(**52.7% to 73.2%**)** but the number of those willing to consider it had decreased from 52.7% to 23.9%^3^. Others have suggested that concerns about pain may be an important factor in women declining the offer of ECV, making up to 30% of patients favour C-Section instead^4,5^.

Indeed, ECV is a procedure associated with a moderate degree of pain^4,6–8^. This was exemplified by a study which demonstrated median pain scores of 5.7 **(**IQR 2.7–6.8**)** in women undergoing ECV on a 10-point visual analogue scale^8^. In addition, significant improvements to success rates of the procedure have been observed when patients have adequate analgesia^6,7,9^. In addressing this, various modalities of pain relief have been trialled including regional anaesthesia, hypnosis, and systemic opioids^9^. All of these are either time consuming or relatively invasive. Furthermore, none of them are particularly appealing to women.

Virtual reality **(**VR**)** is an increasingly promising and affordable technological medium that is used to create simulated scenarios in which users are immersed and able to interact with the virtual environment **(**VE**)** through multisensorial stimulation^10^. There has been increasing interest in assessing its analgesic potential in various fields of medicine, especially since VR has demonstrated clinical efficacy in pain reduction whilst being well-tolerated by patients in a variety of settings e.g. burn wound dressing, venepuncture, and dental treatments^11–16^. Most recently, Frey *et al*. demonstrated significant reductions in pain for women undergoing labour whilst using VR^17^. On top of this, meta-analyses performed by Chan *et al*. and Mallari *et al*. have further substantiated this analgesic effect too^13,18^. To date, however, there has been no investigation of the use of VR in women undergoing ECV.

The precise mechanisms behind the analgesic effects of VR are still under scrutiny by researchers, though several theories have been proposed. For one, it is believed that VR primarily functions as a distraction mechanism as a consequence of its immersive nature^14^. This is based on the premise that the brain has a finite capacity for attention towards pain which can be redirected to attenuate the degree of pain it perceives^19^. Alternatively, it has also been suggested that VR can evoke neurophysiological changes in the pain matrix of the brain thereby dampening its sensitivity to the sensation as well^14,20,21^.

### Objectives

Given the potential of VR to facilitate non-pharmacological analgesia, the question of its utility in reducing procedural pain during ECV merits consideration. This prompted us to design a pilot study to evaluate the use of VR as an analgesic during ECV. The objective of this pilot was to assess feasibility of concept and lay the foundations for an adequately powered randomised controlled trial **(**RCT**)** to test the performance of VR against the standard of care for all women undergoing ECV.

The primary outcomes of interest for the study involved assessing between group differences in pain scores, anxiety scores, physiological parameters [heart rate **(**HR**)**, systolic blood pressure **(**SBP**)** and diastolic blood pressure **(**DBP**)**], women’s acceptance/feedback of the procedure, and side effects encountered between the intervention **(**VR**)** and control **(**no VR**)**.

The secondary outcomes were exploring associations between patient factors and the levels of pain encountered during ECV.

## Methods

### Trial design

The study was an open label randomised controlled pilot study in 50 women. The trial had the approval of the Monash Health Human Research Ethics Committee **(**HREC/18/MonH/413**)**. This trial was also registered on the Australia-New Zealand Clinical Trials Registry **(**ACTRN12618001004257p; registration date: 14/06/2018**)** and was performed in accordance with Good Clinical Practice. Women were recruited via convenience sampling at the time of their attendance for their ECV. Written informed consent was obtained prior to the procedure.

Prior to commencement of the ECV, women were provided with questionnaires to assess their pre-procedural disposition towards pain and anxiety using 101-point numerical rating scales **(**NRS**)**. This was followed by recording the participant’s demographic data and their physiological parameters, heart rate **(**HR**)** and non-invasive blood pressure **(**NIBP**)**. Following this, patients were administered terbutaline **(**250 micrograms**)** subcutaneously as a tocolytic.

The randomisation sequence was generated using Microsoft Excel 2018 with a 1:1 allocation using random block sizes of 10 and was maintained as an electronic list by AN who was independent of the trial. Following successful recruitment, AN was called for allocation consignment. Based on this, patients had either the intervention **(**VR**)** or standard care **(**control**)** administered to them prior to the commencement of the ECV. Standard care entailed no provision of any form of analgesia to the patient. No blinding was performed as both patients and investigators were aware of the intervention administered, given the nature of VR therapy. However, at the time of completing the pre-procedure questionnaire, patients were unaware of their assigned group.

For the procedure, the women were placed on a flat bed with their heads elevated at a 20-degree angle with a pillow underneath. The ECV was then carried out by one of two skilled operators **(**RK/PN**)**, both of whom had each performed more than 500 ECVs.

Following the procedure the number of attempt**(**s**)** and duration of ECV were recorded. An attempt was defined to have commenced when the operator introduced axial force to the maternal abdomen and to have ended when the applied force was ceased. The clinician was also asked to classify the procedure as easy, moderate or difficult utilising their experience with similar procedures in the past.

Post-ECV physiological parameters were also recorded for all women within 5  minutes after the procedure. Women from both groups were then invited to complete a questionnaire evaluating their pain **(**NRS**)**, ECV experience, and side effects. Side effects screened for included dizziness, nausea, vomiting, tremulousness, and flushing.

### Participants

Eligible women were those with a singleton pregnancy and an ultrasound confirmed breech presentation at the time of the ECV.

Clinical exclusion criteria included women with multiple pregnancy, a history of prior uterine surgery, uterine abnormalities, contraindications to vaginal delivery, maternal cardiovascular disease, severe hypertension, pre-labour rupture of membranes, placental abruption, fetal anomaly, and intra-uterine fetal death. Technology-related exclusion criteria encompassed prior history of sensitivity to VR technology, motion sickness, vertigo, seizures, epilepsy, and active nausea and vomiting.

### Study settings

The study was conducted at the Monash Medical Centre, Clayton in Melbourne, Australia from July 2018 to March 2019. Monash Medical Centre is a university teaching hospital providing tertiary level obstetric care.

### Intervention

For this trial, virtual reality content **(**VRC**)** was administered to the participant for the duration of the ECV through a head-mounted display **(**HMD**)**, Samsung Gear VR **(**Samsung, San Jose, California**)**, in combination with a Samsung Galaxy S8 smartphone. The VRC for the trial, “Sky Lights”, was an active form of VR custom designed for the study by ALO VR **(**Singapore**)**. Active VRC was selected as it allows interaction through user input, encouraging a greater level of immersion and presence that can reduce the perception of noxious stimuli^22–24^.

In Sky Lights, the user is placed lying down in a quiet field, staring at a starry night sky with several unlit Chinese lanterns floating gently above. By focusing their gaze on a lantern, the user is able to set it alight, causing the lantern to rise upwards and away. Occasionally, as a reward for continued participation; a lit lantern will either set off a series of fireworks or form Lantern Festival shapes such as a dragon or a giant fish. Relaxing background music is also played to provide auditory stimulation. For this trial, user control was achieved by head tracking and lanterns were lit through triggers on either a Bluetooth hand controller or touchpad on the HMD, based on user preference. Orientation to the device and instructions required approximately 60  seconds and the procedure itself only commenced once the headset was secured onto the patient and verbal confirmation was received that the game had started.

For the control group, no analgesia was administered as per the standard protocols. Patients in both groups were counselled that they could terminate the procedure should the pain become overwhelming.

### Sample size

A sample size of 25 women per arm was chosen, based on recommendations within a review by Whitehead *et al*. that focused on pilot study design and power^25^. These recommendations were aimed to optimise pilot and main trial recruitment when the standardised effect size of the main trial is uncertain but can still be approximated. A conservative small standardised effect size **(**Cohen’s **𝛿** = 0.2**)** was implemented for this purpose, as demonstrated by similar VR studies in differing populations in the context of VR facilitating acute analgesia^15^. This was to generate data to inform a future pivotal RCT with a Type 2 error of 10% and Type 1 error of 5%.

### Statistical methods

Raw data for the numerical variables in the study were explored for distribution using the Shapiro-Wilk test in tandem with visual plot analysis. Approximately normally distributed data was expressed as mean **(**±SD**)** and skewed data was expressed as median **(**IQR**)**. Categorical variables were expressed as a percentage.

Key baseline characteristics and clinical data were presented as descriptive statistics for the study population, intervention and control groups. Differences between intervention and control groups were explored using hypothesis testing.

For the primary outcomes, the change in physiological parameters variables **(**ΔSBP, ΔDBP and ΔHR**)** pre- and post-procedure were computed by the following formula **(**Δparameter = post-procedure parameter -pre-procedure parameter**)**. Hypothesis testing was subsequently carried out to examine between group differences between intervention and control groups for the variables: pre-procedural anxiety, pre-/post-procedural pain, pre-/post-procedural physiological parameters and their fluctuation **(**Δparameter**)**, as well as responses to the questionnaires.

With respect to hypothesis testing for the primary objectives, depending on the distribution, continuous variables were compared using either the independent samples t-test or Mann-Whitney U test. Additionally, the variables: post-procedure pain and change in physiological parameters **(**Δparameter**)**; were compared using an analysis of covariance **(**ANCOVA**)** omnibus test to account for the covariates, pre-procedural anxiety and pre-procedural pain. Categorical data was compared using either the χ^2^ test of homogeneity or z-proportion test. For all tests, the null hypothesis was that there was no difference between the intervention and control group in terms of mean/median/proportion.

For the secondary outcomes, bivariate correlation was implemented to measure the strength of association between variables of interest and the pain scores reported by the patient during the procedure. The null hypothesis was that there was no association between the variables in the population.

For all statistical tests, the assumptions of the test were met and testing carried out was two tailed. Furthermore, statistical significance was set at an alpha level of p < 0.05.

Statistical analysis was completed using SPSS v25.0.

## Results

### Patient characteristics

**Figure 1:**
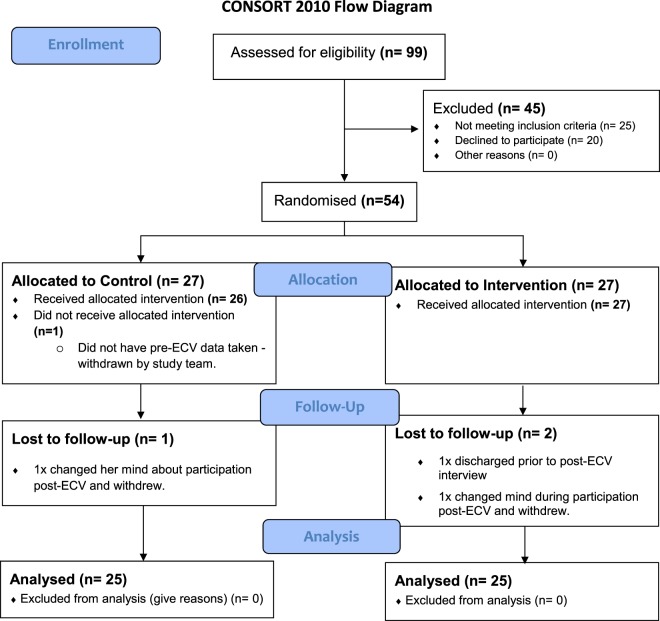
CONSORT 2010 Flow Diagram.

54 women were enrolled into the study. 50 women completed it **(**Fig. 1**)**. 25 women were randomised to the intervention group and 25 women were randomised to the control group. The baseline data and clinical characteristics for the study sample, intervention and control groups are presented in Table 1. There were no significant differences between both control and intervention groups.

**Table 1:** Baseline patient characteristics.

Parameters	Results for entire sample **(**n = 50**)**	Results for intervention **(**n = 25**)**	Results for control **(**n = 25**)**	p-value
Age **(**years**)**	31.56 **(**±5.48**)**^	32.10 **(**±5.50**)**^	31.01 **(**±5.51**)**^	0.49^+^
Gestational age **(**weeks**)**	37.21 **(**1.14**)**	37.43 **(**1.29**)**	37.00 **(**1.22**)**	0.75^
BMI **(**kg/m^2^**)**	28.31 **(**4.28**)**	28.82 **(**5.99**)**	27.34 **(**4.26**)**	0.26^
**Parity status**				
Primiparity	20 **(**40%**)**	11 **(**55.0%**)**	9 **(**45.0%**)**	0.56^#^
Multiparity	30 **(**60%**)**	16 **(**53.30%**)**	14 **(**46.70%**)**	0.56^#^
AFI level **(**cm**)**	13.00 **(**6.77**)**	15.00 **(**8.00**)**	12.00 **(**6.35**)**	0.05^
**Placental location**				
Anterior	7 **(**18%**)**	3	4	0.69~
Fundal	30 **(**75%**)**	16	14	0.65~
Posterior	3 **(**7%**)**	1	2	0.56~
Duration of ECV **(**s**)**	530 **(**749.50**)**	623 **(**722.00**)**	439 **(**751.50**)**	0.20^

^^^Mean **(**±SD**)**, Median **(**IQR**)**, ^+^Independent samples t- test, ^Mann Whitney U test, *statistically significant **(**p < 0.05**)**, ^#^Chi square test, ^~^z- test of proportions, BMI- Body Mass Index, ECV- External Cephalic Version, AFI-Amniotic Fluid Index.

### Pre-intervention results

Table 2 presents the baseline participant characteristics prior to the ECV. The pain anticipated towards the ECV procedure was significantly higher in the intervention group than the control group [Median **(**IQR = 70 **(**23**)** vs 50 **(**0.5**)**; p = 0.03]. Otherwise, there were no significant differences between both groups in relation to their pre-procedural anxiety levels, physiological parameters or attitudes towards the ECV procedure.

**Table 2:** Patient responses to pre-procedure questionnaires and physiological parameters prior to ECV.

Parameters	Results for entire sample **(**n = 50**)**	Results for intervention **(**n = 25**)**	Results for control **(**n = 25**)**	p-value
Rate the level of pain you anticipate with procedure **(**mm**)**	50 **(**20**)**	70 **(**23**)**	50 **(**0.75**)**	0.03*
Rate the level of anxiety you feel about the procedure **(**mm**)**	50.49 **(**±23.98**)**	56.33 **(**±17.71**)**	42.81 **(**±29.17**)**	0.12^+^
HR pre-procedure **(**bpm**)**	84 **(**19**)**	88 **(**14.50**)**	80 **(**19**)**	0.25^
SBP pre-procedure **(**mmHg**)**	125 **(**20**)**	125 **(**10**)**	125 **(**18**)**	0.23^
DBP pre-procedure **(**mmHg**)**	80 **(**15**)**	79.6 ± 8.15	81.6 ± 8.86	0.41^+^
**Do you think ECV is a painful procedure?**				
Yes	37	21 **(**84.0%**)**	16 **(**64.0%**)**	0.11^#^
No	13	4 **(**16.0%**)**	9 **(**36.0%**)**	0.11^#^
**Have you ever experienced an episode of depression in your life?**				
Yes	14	6 **(**24.0%**)**	8 **(**32.0%**)**	0.53^#^
No	36	19 **(**76.0%**)**	17 **(**68.0%**)**	0.53^#^
**Are you anxious about the ECV?**				
Yes	37	21 **(**84.0%**)**	16 **(**64.0%**)**	0.11^#^
No	13	4 **(**16.0%**)**	9 **(**36.0%**)**	0.11^#^

^Mean **(**±SD**)**, Median **(**IQR**)**, ^+^Independent samples t-test, ^^^Mann-Whitney U test, *statistically significant **(**p < 0.05**)**, ^#^Chi square test, ^~^z-test of proportions.

### Post-intervention results

Table 3 presents the post-intervention results.

**Table 3:** Patient responses to questionnaires post-procedure, post-ECV physiological parameters and comparison with pre-ECV observations.

Parameters	Results for entire sample **(**n = 50**)**	Results for intervention **(**n = 25**)**	Results for control **(**n = 25**)**	p-value
Pain during procedure **(**mm**)**	55.20 **(**±25.14**)**	60.68 **(**±21.10**)**	49.76 **(**±28.00**)**	0.17^+^
SBP post-procedure **(**mmHg**)**	121.96 **(**±13.40**)**	122.72 **(**±13.77**)**	121.20 **(**±13.25**)**	0.69^+^
ΔSBP **(**mmHg**)**	−3.50 **(**15**)**	−5.00 **(**12.50**)**	0 **(**17.50**)**	0.57^
DBP post-procedure **(**mmHg**)**	72.00 **(**±9.90**)**	72.20 **(**±10.71**)**	71.80 **(**±9.23**)**	0.89^+^
ΔDBP **(**mmHg**)**	−8.60 **(**±11.00**)**	−9.40 **(**±10.34**)**	−7.80 **(**±11.73**)**	0.61^+^
HR post-procedure **(**bpm**)**	91.94 **(**±12.23**)**	90.84 **(**±11.56**)**	93.04 **(**±13.0**)**	0.530^+^
ΔHR **(**bpm**)**	4.91 **(**±14.31**)**	2.16 **(**±12.41**)**	7.63 **(**±15.78**)**	0.190^+^
Procedural success	39 **(**78%**)**	20 **(**80%**)**	19 **(**76%**)**	0.73^#^
**Procedural difficulty**				
Easy	18 **(**36.0%**)**	9 **(**36.0%**)**	9 **(**36.0%**)**	0.94^#^
Moderate	17 **(**34.0%**)**	9 **(**36.0%**)**	8 **(**32.0%**)**	
Difficult	15 **(**30.0%**)**	7 **(**28.0%**)**	8 **(**32.0%**)**	
Number of attempts	2 **(**2**)**	2**(**2**)**	2**(**2**)**	0.60^
Duration of ECV **(**s**)**	530 **(**749.50**)**	623 **(**722.00**)**	439 **(**751.50**)**	0.20^
How would you rate using the device **(**mm**)**?		75 **(**32.50**)**		
Side effects noted during procedure	13 **(**25.5%**)**	6 **(**24.0%**)**	7 **(**28.0%**)**	0.75^#^
**Would you reconsider your decision to have the procedure based on the pain felt?**				
Yes	10 **(**20%**)**	3 **(**12.0%**)**	7 **(**28.0%**)**	0.16^#^
No	40 **(**80%**)**	22 **(**88.0%**)**	18 **(**72.0%**)**	

^Mean **(**±SD**)**, Median **(**IQR**)**, ^+^Independent samples t-test, ^^^Mann-Whitney U test, *statistically significant **(**p < 0.05**)**, ^**#**^Chi square test, ^~^z-test of proportions, Δchange in pre- and post-intervention parameter.

**Figure 2:**
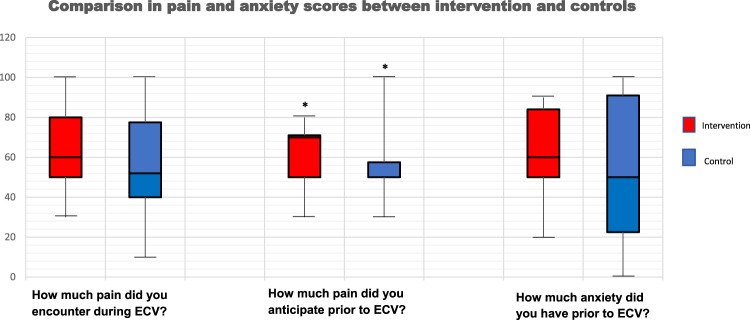
Comparison of pain and anxiety scores between the intervention and control groups pre- and post-procedure. Statistically significant difference was defined as p < 0.05 and is denoted by (*).

For the intervention and control groups, there were no statistically significant differences between the pain score [60.68 **(**±21.1**)** vs 49.76 **(**±28.00; p = 0.17], ECV success rates **(**80% vs 76%; p = 0.73**)**, physiological parameters and change in physiological parameters pre- and post-intervention **(**Fig. 2**)**. When using ANCOVA to correct for anticipated anxiety and pain, the adjusted means for pain scores **(**±standard error**)** between intervention and control were not statistically significantly different [62.0 **(**±4.9**)** vs 55.3 **(**±6.3**)**; p = 0.42**)**]. Similarly, there was no significant differences in ΔSBP [−3.37 **(**2.18**)** vs −1.33 **(**2.76**)**; p = 0.58], ΔDBP [−10.45 vs −10.57; p = 0.98] and ΔHR [−1.10 **(**3.0**)** vs 7.23 **(**3.70**)**; p = 0.09] upon correction.

Side effects were encountered in 25.5% of the study sample but was not significantly different between intervention and controls **(**24% vs 28%; p = 0.75**)**. These included dizziness, nausea, vomiting, tremulousness and flushing.

In the intervention group, 20 women **(**80%**)** indicated that they would use the VR again in a subsequent ECV procedure and 19 **(**76%**)** believed it should be offered as a routine part of the ECV. Twenty-two women **(**88%**)** said that they would recommend it to a friend undergoing ECV.

For our secondary objectives, we carried out bivariate correlation to investigate the association of participant and procedural characteristics with the pain scores reported **(**Table 4**)**. Several variables were statistically significant and demonstrated a moderate correlation [total duration of ECV **(**ρ = 0.37; p = 0.01**)**, level of anxiety felt pre-procedure **(**ρ = 0.36, p = 0.03**)**, procedural difficulty **(**ρ = 0.36; p = 0.01**)** and pain anticipated pre-procedure **(**ρ = 0.40; p = 0.02**)** with pain reported by the women post-ECV.

**Table 4:** Correlation matrix between study variables and level of pain reported by women.

Factor	ρ **(**Spearman’s rho**)**	p value
Age	−0.10	0.51
Gestational Age	0.18	0.22
BMI	0.05	0.71
AFI level	−0.30	0.84
Total duration of ECV	0.37	0.01^*^
Number of attempts at ECV	0.17	0.24^*^
Level of anxiety felt pre-procedure	0.36	0.03^*^
Parity status	0.20	0.17
Placental location	0.08	0.61
Procedural difficulty	0.36	0.01^*^
Pain anticipated pre-procedure	0.40	0.02^*^
Intervention status	0.24	0.10

^*^Statistically significant **(**p < 0.05**)**.

## Discussion

This pilot study provides preliminary evidence of the feasibility of approach and acceptance from mothers for the novel use of VR as an analgesic during ECV. In this context, and as intended *a priori*, we believe there is merit in investigating the ability of VR to facilitate analgesia during ECV through an RCT.

Overall, this pilot did not demonstrate any significant differences in pain scores and physiological parameters between the intervention and control groups. This, however, should be construed in line with the motivations behind pilot studies in general, which is to determine feasibility-of-concept. In addition, this pilot was inadequately powered for hypothesis testing, thereby limiting the interpretation and generalisability of the results. Furthermore, the intervention group in our study had significantly higher pain anticipation in contrast to the control, which is evidenced by the moderate correlation between anticipated pain and anxiety scores pre-procedure with post-procedural pain scores. These anticipatory elements have been previously linked with increased perceived pain scores in several studies which may have contributed to the higher non-significant pain trend in our intervention group^26–28^.

Importantly, the findings of our pilot are in stark contrast to several other higher quality studies aimed at assessing the utility of VR during acute pain. To date, several systematic reviews and experimental studies have demonstrated the analgesic efficacy of VR for management of acute pain^11,13,14,29–31^. In particular, the recent systematic review by Chan *et al*. deserves mention as they demonstrated through their meta-analysis a standardised mean difference of −0.49 **(**95% CI −0.84 to −0.41, p < 0.01**)** in pain reduction with the use of VR in acute pain^13^. To further reiterate this, the prospective cohort study by Tashjian *et al*. remains informative as well, demonstrating a 24% reduction in pain scores with VR use and a number needed to treat **(**NNT**)** of 4 to achieve an episode of reduced pain^15^. These findings suggest that VR is efficacious in facilitating acute analgesia, with the added advantages of being safe and cost-effective as well. Considering the shortcomings of a pilot design and the available evidence on VR’s analgesic efficacy, it would be unwarranted to discount the utility of VR in ECV without a formal evaluation.

One of the cornerstones of healthcare improvement, as formulated by the Institute of Healthcare Improvement, is with respect to the enhancement of patient experience^32^. In this regard, the findings of our pilot remain promising and in-line with other pregnancy-related studies which have demonstrated a high level of maternal satisfaction and acceptability for VR, with approval ratings between 77–82%^17,33^. This alludes to women being interested in and welcoming the addition of the technological medium into their pregnancy care. On this basis, assessing the utility of VR in enabling a better ECV experience as well as promoting greater procedural acceptance is an aspect which deserves formal evaluation since it could potentially influence future clinical management in an area where there is currently no analgesic standard of care^34^.

Our pilot also revealed side effects in approximately 25% of women. This rate is paradoxically higher in comparison to similar VR studies which have demonstrated side effects from the intervention to be between 0–5%^15,35,36^. However, these rates were similar in both, the control and intervention groups, leading us to attribute this phenomena to the terbutaline employed in the study. Furthermore, despite this higher rate of side effects, mothers continued to partake in the VR procedure, further outlining the acceptability of the intervention.

It was found that this study has several limitations. Firstly, being a pilot, it was inadequately powered to detect a difference in the pain scores and physiological parameters. However, the basis of this study was to determine feasibility of concept, safety of VR, and to elicit participant feedback. As such, the study remains valuable in informing a future multicentre RCT on the issue.

Secondly, the utilisation of terbutaline for ECV is a factor which merits consideration in the context of the impact it may have had on the findings of our pilot. Terbutaline administration is associated with adverse side effects of the sympathetic activation, such as tachycardia, hypertension, nausea, vomiting, tremulousness, and flushing. These overlap considerably with the side effects screened for in this study and those implicated with VR use. This could have introduced measurement bias into the study as the adverse side effects detected in both groups may have been a result of the drug side effects. In addition, the terbutaline could have affected the physiological parameters of the patient as well thereby limiting their usefulness as a physiological measure of pain. Future studies should utilise more objective measures of physiological pain, such as electrodermal activity measurements, to negate this drug-related effect.

Next, due to the recruitment being sourced from a single site via convenience sampling, there is potential for spectrum bias to have been introduced into the study. This could be addressed by performing a multi-centre RCT as well.

Lastly, taking the non-significant findings of the trial into account, further information regarding the individual experience of the virtual reality interaction would have provided a more comprehensive understanding of our results. This includes elements such as the sense of presence in the virtual environment, appeal of the experience and familiarity with VR utilisation. Like several clinical evaluations of VR in the setting of acute pain, this was, unfortunately, not an element of our pilot evaluation. The pilot, however, highlighted the significance of acquiring such data and this will be incorporated into our larger scale evaluation to obtain a broad understanding of factors which may affect the analgesic properties of VR.

## Conclusions

With rising evidence supporting the clinical efficacy of VR as a form of non- pharmacological analgesia, varied indications for its use are increasingly being explored. This pilot serves as preliminary evidence for the feasibility, safety and acceptance of its utilisation during ECV. This serves to inform future controlled studies on the issue to systematically investigate its utility during the procedure.

## Data Availability

The datasets generated during and/or analysed during the current study are not publicly available to protect individual patient information and data but are available from the corresponding author on reasonable request.
